# *In vivo* Assessment of Antioxidant and Wound Healing Improvement of a New Schiff Base Derived Co (II) Complex in Rats

**DOI:** 10.1038/srep38748

**Published:** 2016-12-13

**Authors:** Rashd. M. El-Ferjani, Musa Ahmad, Summaya M. Dhiyaaldeen, Farah Wahida Harun, Mohamed Yousif Ibrahim, Hoyam Adam, Bohari Mohd. Yamin, Mazen M. Jamil Al-Obaidi, Rami Al Batran

**Affiliations:** 1 Faculty of Science and Technology, Universiti Sains Islam Malaysia, 71800, Nilai, Negeri Sembilan DK, Malaysia; 2 Department of Microbiology, Faculty of Medicine, University of Duhok, 78 Kurdistan, Iraq; 3 Department of Pharmacy, Faculty of Medicine, University of Malaya, 50603 Kuala Lumpur, Malaysia; 4 Department of Chemistry, Faculty of Science, University of Malaya, 50603 Kuala Lumpur, Malaysia; 5 School of Pharmacy, Ahfad University for Women (AUW), 167 Omdurman, Sudan; 6 School of Chemical Sciences and Food Technology, Universiti Kebangsaan Malaysia, 43600, Bangi, Selangor D.E., Malaysia; 7 Medical Microbiology Department, Faculty of Medicine, University of Malaya, 50603 Kuala Lumpur, Malaysia; 8 Institute of Research Management & Monitoring, Deputy Vice Chancellor (Research and Innovation), University of Malaya, 50603 Kuala Lumpur, Malaysia

## Abstract

Co (II) complex (CMLA) was investigated to evaluate the rate of wound healing in rats. Animals were placed into four groups: gum acacia, Intrasite gel, 10 and 20 mg/ml of CMLA. Wounds were made on the dorsal neck area, then treated with Intrasite gel or CMLA; both of these treatments led to faster healing than with gum acacia. Histology of the wounds dressed with CMLA or Intrasite gel displayed a smaller scar width, required less time to heal and showed more collagen staining and fewer inflammatory cells in comparison to wounds dressed with the vehicle. Immunohistochemistry for Hsp70 and TGF-β showed greater staining intensity in the treated groups compared to the vehicle group. Bax staining was less intense in treated groups compared to the vehicle group, suggesting that CMLA and Intrasite gel provoked apoptosis, responsible for the development of granulation tissue into a scar. CD31 protein analysis showed that the treated groups enhanced angiogenesis and increased vascularization compared to the control group. Furthermore, a significant increase in the levels of GPx and SOD and a decrease in MDA were also observed in the treated groups. This results suggest that CMLA is a potentially promising agent for the wounds treatment.

In developing countries, the treatment of wounds and burns poses a significant problem. Wound healing is a natural, dynamic and interactive mechanism that involves various components such as soluble mediators, the extracellular matrix, blood cells and parenchymal cells. Wound healing involves three stages, namely the inflammatory stage, the proliferative stage, and the remodeling stage. The remodeling stage is the most important phase as it determines the strength and manifestation of the healed tissue. Wound healing is a complex series of interrelated events that are mediated through these phases by a wide range of chemically coordinated cellular processes as well as hormonal influences^1^. Wound healing commences at the time an injury occurs and progresses for varying lengths of time based on the extent of the injury. Subsequently, after an injury, an inflammatory response is triggered and cells located under the dermis increase the synthesis of collagen production and regenerate epithelial tissue^2^. Wound repair is an immune-mediated physiological mechanism and in which the skin repairs itself after an injury^3^. However, wound healing can have severe complications that invoke high costs for therapy; therefore, it is necessary to develop more efficacious methods to heal wounds and burns in order to improve healing and reduce the cost involved^4^.

Schiff bases are considered to be an important category of organic agents in the field of medicinal chemistry^5^,^6^, and the study of new Schiff base complexes with therapeutic efficacy has drawn the interest of many researchers^7^. Schiff bases, along with their complexes, are multipurpose agents resulting from the reaction of an amino compound with a carbonyl agent. These materials are extensively utilized for industrial purposes as well as for biological applications due to their antifungal, antibacterial, antiviral, antiproliferative, antimalarial, anti-inflammatory, antipyretic activities^8^. Cobalt has been found to be a core factor in the active site of vitamin B12 (cobalamin), which indirectly controls the production of DNA. Cobalamin functions as a carrier ligand and contributes to the accumulation of metallodrugs in cancer cells, since proliferating cells demand higher quantities of vitamin B12 as compared to resting cells^9^. Cobalt complexes that have undergone structural characterization are used as hydrolytic agents for DNA cleavage^10^,^11^ and have been shown to possess antitumor, anti-proliferative, antimicrobial, antifungal, antiviral and antioxidant activity^12^.

Recently, a Co (III) Schiff base complex (Doxovir) was successful in a phase II clinical trial for the treatment of herpes simplex virus^13^. This study was designed to investigate the wound healing potential of applying a new Co (II) complex with a Schiff base derived from 4-dimethylaminobenzaldehyde with L-asparagine in rats.

## Methodology and Materials

### Chemicals, reagents and drugs

**Figure 1:**
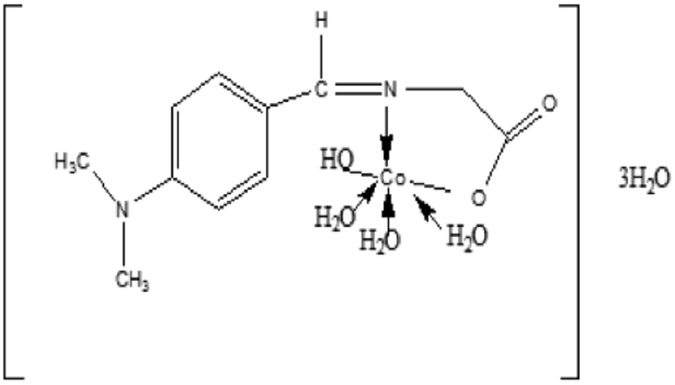
Chemical structure of CMLA [Co L (H_2_O)_3_(OH)]3H_2_O.

All chemicals and reagents were purchased from Sigma (Sigma Aldrich, Germany) and used without further purification. University Malaya Medical Centre Pharmacy supplied the Lignocaine HCl (Delta Veterinary Laboratory PTY LTD, NSW 20011) and Intrasite gel (a product of Smith and Nephew Healthcare Limited). Intrasite gel can be used at every stage of the wound healing process^14^. CMLA (Fig. 1) was dissolved as described by Shetty *et al*. with some alterations using the vehicle (gum acacia) in normal saline^15^.

### Formation of the Schiff base

The Schiff base formed from 4-dimethylaminobenzaldehyde and L-asparagine was prepared by the addition of 25 ml of a 4-dimethylaminobenzaldehyde solution in ethanol (1.49, 0.01 mol) to the same volume of an ethanolic solution of L-asparagine (0.01 mol). This was then heated to reflux for two hours. The end product was collected by filtration, cleaned with ethanol a few times and recrystallized using boiled ethanol^16^.

### Formulation of Schiff base metal complexes

First, the metal complexes were processed. Metal complexes were formulated by mixing 25 ml of the ethanolic solution of the prepared Schiff base (0.01 mol) and aqueous ammonia. The solution was then subjected to reflux for 2 hours and subsequently filtered. The metal complexes that had been filtered were then washed with hot ethanol until they turned colorless. Air drying the final product over phosphorus penta-oxide constituted the final step^16^. Elemental analysis and spectral characterization for the ligand and its metal complex are presented in Table 1.

### Ethical issues

All the procedures were performed in accordance with the approved guidelines of the Institutional Animal Ethical Committee of the University of Malaya [Ethics certificate no. (PM/27/7/2014/RAB (R)]. Healthy Sprague-Dawley rats with an average body weight of 230–250 g were provided by the Animal House, Faculty of Medicine, University of Malaya, Kuala Lumpur. The rats were fed a standard diet and tap water *ad libitum*, and were kept separately in cages with wide-mesh wire bottoms to prevent coprophagia throughout the experiment.

### Acute toxicity test and experimental animals

To determine the safe dosage of CMLA, an acute toxicity study was conducted. Thirty-six Sprague Dawley rats (18 males and 18 females) were arranged into three groups and treated with vehicle (0.5% CMC, 5 ml/kg), or given 500 or 1000 mg/kg of CMLA (5 ml/kg) orally. The animals were deprived of food overnight before dosing. After dosing, food was withdrawn for an additional 3 to 4 h. The subjects were monitored for 48 h for toxicological symptoms. Death of the animals was also recorded throughout the experiment. On the 15^th^ day, the animals were overdosed with xylazine and ketamine. Histological, hematological and serum biochemical parameters were assessed following standard methods^17^,^18^.

### Experimentally inflicted wounds

Male Sprague Dawley rats were randomly segregated into four groups of 12 rats each and caged individually. The animals were anesthetized with 0.09 ml of ketamine i.m. (30 mg/kg, 100 mg/ml) and 0.01 ml of Xylazil i.m. (3 mg/kg, 100 mg/ml) prior to the creation of wounds. The dorsal neck of each rat was shaved using an electrical shaver, disinfected with 70% ethanol and injected with 1 ml of lignocaine HCl 2% 20 (mg/ml) as a local anesthetic. Using a circular stamp (2 cm in diameter), an impression was made in the dorsal thoracic region, 1 cm away from the vertebral column and 5 cm away from the ear, then an excision wound was created by cutting out the full thickness of skin that had been marked by the stamp^4^.

### Topical application of vehicle

The wounds of Group 1 rats were treated with 0.2 ml of gum acacia in normal saline, twice a day; this served as the control group. The wounds of Group 2 rats were treated with 0.2 ml Intrasite gel twice a day and served as a reference standard control. The wounds of rats in Groups 3 and 4 were treated with 0.2 ml of CMLA (10 or 20 mg/kg, respectively) twice a day. The wounds were observed daily throughout the experiment and animals were euthanized when complete wound healing took place.

### Estimation of the rate of wound healing (wound closure)

Progressive changes in wound healing were assessed by tracing the wound area on transparent tracing paper on days 1, 5, 10 and 15 post-wound surgery. Transparent paper was laid over the wound and the wound limits were traced it on it using a permanent marker. The wound area was then quantified using graph paper. The tracing paper was placed on a sheet of graph paper (2 mm^2^) and the number of squares within the wound area was counted^19^. The percentage of wound contraction was measured, using the initial area and the areas determined on days 1, 5, 10 and 15 using the following formula^15^:


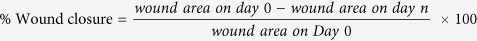


where n = number of days (5, 10, 15) days that the healed area was read.

After healing was complete, the healed skin was excised under anesthesia and placed in PBS for homogenization or in 10% buffered formalin for histological evaluation.

### Preparation of tissue homogenates

Wound tissues were rinsed with ice-cold saline as the initial step. Samples were then homogenized using a homogenizer (Polytron, Heidolph RZR 1, and Germany). The homogenate was processed on ice at a concentration of 10% (w/v) in potassium phosphate buffer (50 mM, pH 7.8) containing mammalian protease inhibitors. The homogenates were then centrifuged for 30 minutes at 4,500 rpm and 4 °C.

### Lipid peroxidation and antioxidant activity in granulation tissue

Wound tissue collected and tissue homogenates were prepared for the measurement of antioxidant enzyme activity, i.e. glutathione peroxidase (GPx) and superoxide dismutase (SOD). The level of lipid peroxidation was then assessed by determining the level of malondialdehyde (MDA) using a commercial kit according to the manufacturer’s instructions (Cayman, Michigan, USA).

### Histological examination

Sample pieces of skin were taken from healed injuries from each rat and placed in 10% buffered formalin solution to facilitate histopathological studies. Healed skin samples were cut at a thickness of 5 μm and stained with hematoxylin and eosin (H&E). Histopathological changes were then observed and recorded.

### Masson’s trichrome staining

Wound tissue samples were stained for collagen using Masson’s Trichrome (Sigma, USA). All sample slides were inspected using a light microscope and images were captured using a Nikon microscope (Y-THS, Japan).

### Immunohistochemical staining

Using a hot air oven, tissue section slides were heated at 60 °C for about 25 minutes (Venticell, MMM, Einrichtungen, Germany). The tissue sections were de-paraffinized in xylene and rehydrated with a graded alcohol series. The sample slides were boiled in 10 mM sodium citrate buffer in a microwave for antigen retrieval. Next, 0.03% hydrogen peroxide containing sodium azide was applied for 5 minutes to block endogenous peroxidase. Tissue sections were then washed gently with wash buffer, then incubated with biotinylated primary antibodies against Hsp70 (1:500), Bax (1:200) and TGF-β (1:500) (Abcam, UK) for 15 minutes. The tissue sections were washed gently with wash buffer and placed in a buffer bath. Next, the slides were placed in a humidified chamber with a sufficient amount of streptavidin-HRP (streptavidin conjugated to horseradish peroxidase in PBS containing an anti-microbial agent) for 15 minutes. Diaminobenzidine-substrate-chromagen was added to the tissue sections and incubated for another 5 minutes. This was followed by washing and counterstaining with hematoxylin for 5 sec. The tissue sections were then dipped in weak ammonia (0.037 mol/L) 10 times and washed with distilled water. Positive immunohistochemical findings were observed by the formation of brown staining and assessed by light microscopy.

### Morphometric analysis

Images for analysis which were captured (Nikon Y-THS, Japan) by two experienced observers in an independent fashion, blinded to the control and experimental groups. The mean percentage of inflammatory cells and fibroblasts were determined in high power fields (HPF) at 40× magnification. For collagen, Bax and Hsp70 stained areas on each slide were examined microscopically under (HPF) at 100× magnification in each experimental group and the control group using an optical image analyzer (Imager Plus 4.5, Media Cybernetics, Silver Spring, MD). The findings are reported as the mean percentage of cells or stained area. A value of P < 0.05 was considered statistically significant^20^.

### Western blotting

Homogenized samples of dermal wounds were separated on 4–20% sodium dodecyl sulfate (SDS-PAGE) gels and the proteins were transferred to polyvinylidene fluoride (PVDF) membranes. Briefly, the membranes were blocked with 5% non-fat milk followed by incubation with a primary antibody recognizing platelet endothelial cell adhesion molecule (PECAM-1) or cluster of differentiation 31 (CD31) antibody (ab124432; 1:1000 dilution) at 4 °C overnight. The membranes were washed and incubated with HRP-conjugated goat anti-rabbit IgG. Band densities were normalized to the total amount of protein loaded in each well, as determined by densitometry analysis of the PVDF membranes stained with Amersham ECL Prime Western Blotting Detection Reagent (GE Healthcare). The proteins were visualized by chemiluminescence (UVP, Bio Spectrum, USA) and the densities of specific bands were quantified by densitometry using Image J software version 1.37 (NIH, USA)^20^. The housekeeping protein β-actin (1:1000) was used as the loading control.

### Statistical analysis

All findings are reported as the mean ± SEM. Data obtained from these experiments were examined using the Statistical Package for the Social Sciences (SPSS 18, SPSS Inc., Chicago, IL). One-way analysis of variance (ANOVA) and Dennett’s post hoc test for average comparison were utilized. *P* < 0.05 was considered statistically significant.

## Findings

### Acute toxicity study

**Figure 2:**
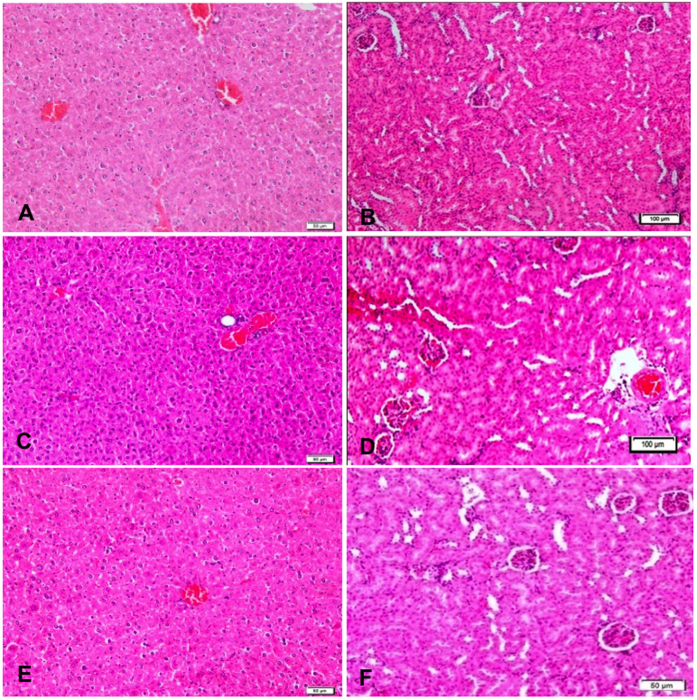
Effect of CMLA on histological sections of the liver and kidney in rats. (**A**,**B**) Rats treated with vehicle. (**C**,**D**) Rats treated with 500 mg/kg of CMLA. (**E**,**F**) Rats treated with1000 mg/kg of CMLA. There is no significant difference in the architecture of the livers (**A**,**C**,**E** 10× magnifications) and kidneys (**B**,**D**,**F** 20× magnifications) between the treated and control groups (H &E stain, (A-F 20× magnifications Hematoxylin and eosin stain).

An acute toxicity study was performed whereby the rats were administered with CMLA at a dose of 500 mg/kg or 1000 mg/kg. The rats were observed for 14 days. The outcomes revealed that all the animals remained alive and did not display any significant signs of toxicity at the administered doses. The clinical observations, histopathology and serum biochemistry data did not reveal any noticeable differences between the treated groups and the control (Fig. 2 and Table 2).

### Evaluation of wound healing

**Figure 3:**
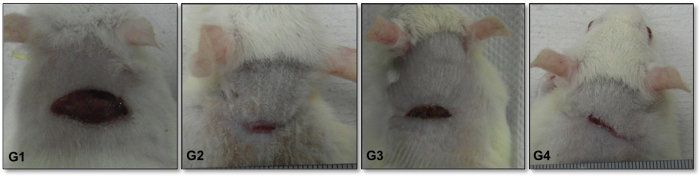
The gross appearance of wound healing on day 15. (G1) Gum acacia-treated group showing incomplete wound healing, (G2) Intrasit gel group showing complete wound healing, (G3) 10 mg/ml of CMLA group showing almost complete wound healing, (G4) 20 mg/ml of CMLA group showing complete wound healing.

The observations revealed that wounds treated with CMLA or with the Intrasite gel revealed some signs of dermal healing and healed relatively faster in comparison with the group that was given the placebo control treatment (gum acacia in normal saline) (Fig. 3 and Table 3). Rats with wounds dressed with the higher CMLA dose of 20 mg/kg demonstrated a wound healing rate equivalent to that of Group 2 (Intrasite gel). Rats that received the low CMLA dose of 10 mg/kg showed a faster wound healing rate than that of Group 1, but healing was slower than the rates observed in Group 2 and Group 4 (Fig. 3 and Table 3).

Table 4 shows the effect of CMLA on the percentage of wound healing over time. The rats treated with a high dose of CMLA (Group 4) had a similar percentage of healing as rats treated with Intrasite gel (Group 2), and better results than the rats treated with the low dose of CMLA (Group 3).

### Histological assessment

**Figure 4:**
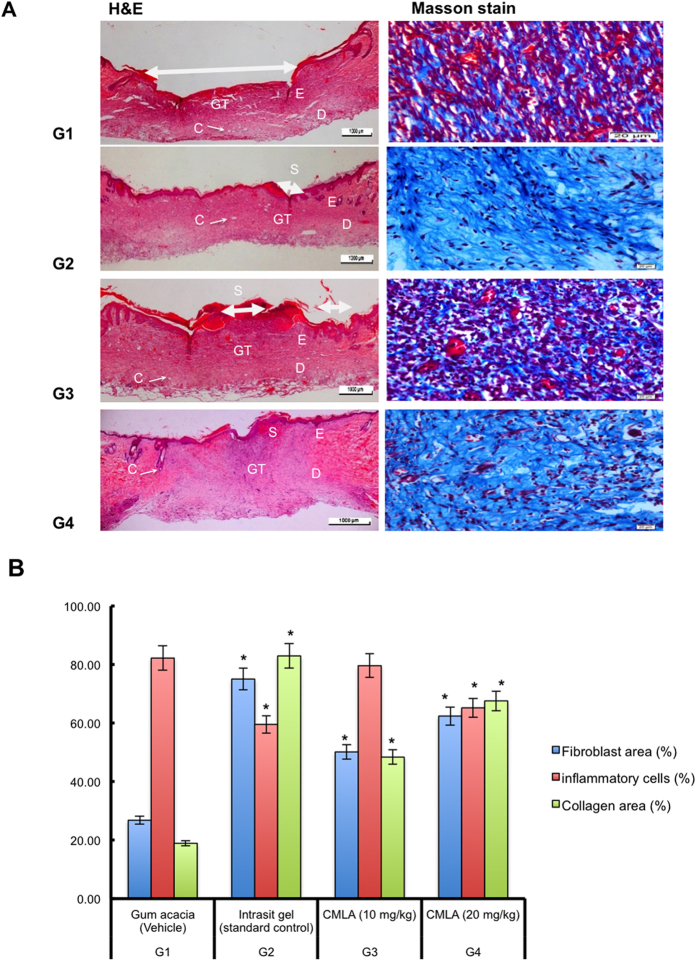
Effect of CMLA on histological section (H&E) and Masson’s Trichrome staining of healed wound on day 15 post-surgery. The arrow showed epithelialization. S-Scab; E-Epidermis; D-Dermis; GT-Granulation tissue; C-Capillaries. (G1) Gum acacia group showing incomplete wound healing enclosure and few degree and alignment of collagen (G2) Intrasit gel group showing complete wound healing enclosure and a greater degree and alignment of collagen (G3) 10 mg/ml of CMLA group showing narrow scar region of wound closure and moderate degree and alignment of collagen. (G4) 20 mg/ml of CMLA group showing complete wound healing enclosure and more degree and alignment of collagen (H&E stain 4×), (Masson’s Trichrome stain 100×). Image analysis was executed using an optical image analyzer (ImagePro Plus 4.5, Media Cybernetics, Silver Spring, MD). Data was expressed as the mean ±SEM (n = 6) and analyzed using one way analysis of variance ANOVA followed by Dunnett’s post hoc test for average comparison on SPSS 18.0. Mean values ± SEM were used. Significance was defined as *p < 0.05 compared to G1.

Microscopic images of H&E stained tissue sections at 4× magnification are displayed in Fig. 4. The microscopic appearance of skin from the gum acacia rats (Group 1) showed a greater scar width at 4× magnification, along with many invasive inflammatory cells, few fibroblasts and blood vessels, and minimal signs of collagen. Skin from animals treated with Intrasite gel and the high dose of CMLA (Groups 2 and 4) showed a reduction in scar width at 4× magnification, fewer inflammatory cells, more fibroblasts and blood vessels, and mild collagen deposition. The skin of rats given the low dose of CMLA (Group 3) showed a reduction in scar width at 4× magnification, with fewer inflammatory cells, more fibroblasts and blood vessels and moderate collagen deposition compared to rats treated with gum acacia (Group 1). However, the healing was not as good as that noted in Group 2 and Group 4. Morphometric image analysis confirmed the effect of CMLA treatment on wound healing in rats.

### Antioxidant enzyme activities and MDA levels in wound tissue

**Figure 5:**
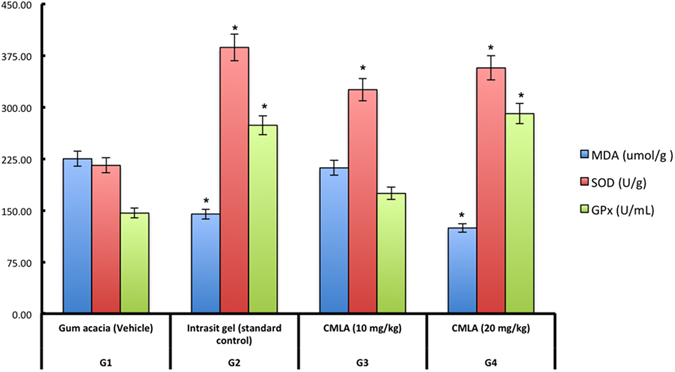
The effects of CMLA on MDA, SOD and GPx in tissue homogenates of dermal wounds rats. (G1) Gum acacia group. (G2) Intrasit gel group, (G3) 10 mg/ml of CMLA and (G4) 20 mg/ml of CMLA group. Statistical Analysis of the data were carried out using one way analysis of variance (ANOVA) and Dennett’s post hoc test for average comparison on SPSS 18.0. Mean values ± SEM were used. Significance was defined as *p < 0.05 compared to Gum acacia.

GPx and SOD activities and MDA levels were measured (Fig. 5). The results show that, in comparison with Group 1, GPx and SOD were significantly increased in Groups 2 and 4. In addition, a notable reduction in the MDA level was observed in the tissue obtained from animals treated with CMLA compared with levels in tissue from the control group.

### Immunohistochemistry

**Figure 6:**
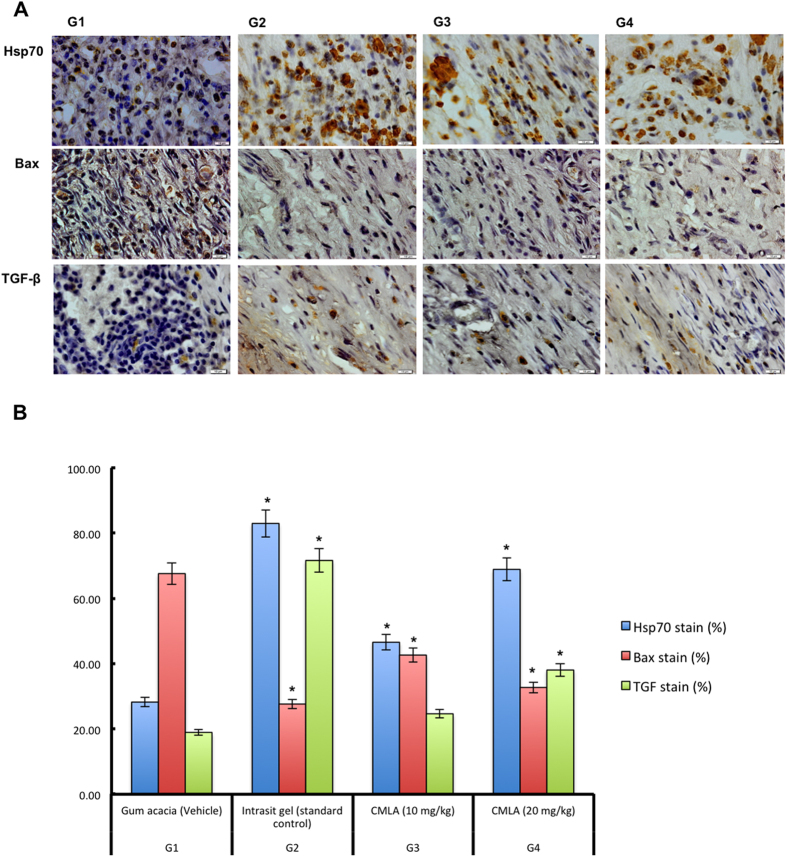
Immunohistochemical staining (Hsp70, Bax and TGF-β) of healed wound section on day 15 post-surgery. (G1) Gum acacia group. (G2) Intrasit gel group, (G3) 10 mg/ml of CMLA and (G4) 20 mg/ml of CMLA group (Magnification 100×). Image analysis was executed using an optical image analyzer (ImagePro Plus 4.5, Media Cybernetics, Silver Spring, MD). Data was expressed as the mean +-SEM (n = 6) and analyzed using one way analysis of variance ANOVA followed by Dunnett’s post hoc test for average comparison on SPSS 18.0. Mean values ± SEM were used. Significance was defined as *p < 0.05 compared to G1.

Bax, Hsp70 and TGF-β staining of the skin from all experimental groups is shown in Fig. 6. Skin tissues from the gum acacia treated group (Group 1) showed more intense Bax staining compared with the groups administered with Intrasite gel and CMLA. The skin from the Intrasite gel and CMLA treated groups (Groups 2, 3 and 4) showed less intense of Bax staining, indicating that the treatment induced apoptosis. These findings indicate that CMLA can accelerate wound healing by increasing apoptosis in damaged skin. On the other hand, skin tissues from the gum acacia treated group (Group 1) showed very little Hsp70 and TGF-β expression, indicating a delay in healing with an increased number of inflammatory cells. However, skin from the Intrasite gel and CMLA treated groups (Groups 2, 3 and 4) showed more Hsp70 and TGF-β expression, indicating an improvement in the healing process with fewer inflammatory cells. Rats treated with CMLA at the doses of 10 and 20 mg/kg showed similar outcomes as Intrasite gel treated rats for both of these parameters.

### Western blotting

**Figure 7:**
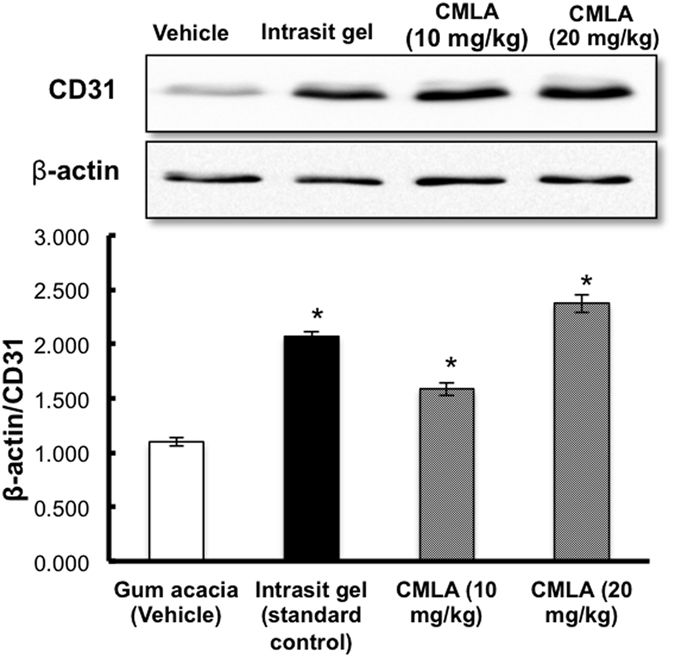
The effect of CMLA on CD31 expression in tissue homogenates of dermal wounds rats. The data were analyzed by density color and displayed as mean ± S.E.M. Significance difference was defined as *P < 0.05 compared to Gum acacia group.

The effect of CMLA on CD31 expression in the tissue homogenates of dermal wounds in the different experimental groups are shown in Fig. 7. Throughout the western blot analysis, we undertook a comparable analysis of CD31 protein in the tissue homogenates of each group where β-actin was used as the loading control. The results show that CD31 expression was significantly greater in rats that received the CMLA (Groups 3 and 4) compared with the gum acacia group (Group 1). Moreover, the expression of CD31 in the Intrasite gel group (Group 2) was significantly higher than in the gum acacia group. These results confirm the ability of CMLA to enhance angiogenesis and increase vascularization.

## Discussion

The elemental analysis data of the Schiff base complex (Table 1) revealed the formation of a 1:1 [M: L] ratio. It was found that the theoretical values were in a good agreement with those identified in the present study. The purity of the Schiff base complex was further studied using the TLC technique and elemental (C, H and N) analysis. The IR spectrum of the L-asparagine Schiff base complex showed a band at 1576 cm^−1^ attributed to ν(C = N) of azomethine; changes in this band indicate its involvement in complexation with metal ions. The infrared spectral results of the same Schiff base complexes showed a band at 1611 cm^−1^, suggesting the existence of COO- groups in the L-asparagine compound. This band appeared in a higher region compared to its original position in the free ligand of 1506 cm^−1^. The same spectrum displayed a broad band at 3465 cm^−1^, attributed to the presence of water molecules during complex formation. The discovery of new bands at 596 cm^−1^ and 678 cm^−1^ that were attributed to ν(M–N) and ν(M–O) vibrations confirmed the involvement of nitrogen and oxygen atoms in coordination with metal ions. The electronic spectrum of the Co (II) complex of the type [Co L (H_2_O)_3_.(OH)].3H_2_O showed two bands at 380 and 520 nm (12666 and 17333 cm^−1^). The first band at 380 nm was due to the ^4^T_1g_ (F) → ^4^T_2g_ (F) transition while the second band at 520 nm was assigned to the 4T_1g_ (F) → ^4^T_1g_ (P) transition. In addition, the ^1^H-NMR spectrum of the ligand showed several characteristic chemical shifts (using DMSO as the solvent): a singlet signal at 8.45 δ ppm corresponding to the hydroxyl proton, a peak at 7.9 δ ppm attributed to the azomethine proton (CH = N-), an aromatic benzene ring at 7.5–7.2 δ ppm and a peak at 3.044 δ ppm attributed to (CH_3_)_2_N. The absence of an –OH proton occurred due to complexation. There was an appreciable change in all other signals in this complex.

Wound healing or wound contracture is important as it contributes to restoring the cellular structure in damaged tissues back to a healthy condition. It is a very complex process involving many dynamic processes, starting with the fibroblastic phase in which the area of the wound shrinks. Next, in the proliferative stage, angiogenesis, collagen deposition, granulation tissue formation, epithelialization and wound contraction occur and small scar tissue forms^21^. The results of this experiment demonstrate that topical application of CMLA improved the rate of wound healing. It was also found that the granulation tissue contained comparatively fewer inflammatory cells, more collagen and increased angiogenesis according to the histology results. These findings support study by Mughrabi *et al*.^22^ in which it was found that wounds treated with Bis[benzyl N′-(indol-3-ylmethylene)-hydrazinecarbodithioato]-zinc(II) (BHCZ) contained fewer inflammatory cells, more collagen and increased angiogenesis in the granulation tissue.

Wound healing may have occurred because of the regulation of collagen expression^23^ and an increase in the tensile strength of the wounds^24^. Healing activity depends on collagen formation and angiogenesis^25^. Collagen is important as it is the core component needed to connect tissues and at the same time act as a framework for newly generated tissues^4^. Angiogenesis is a process whereby the blood circulation in the area of the wound is increased. This improves the transport of nutrients and oxygen, which are crucial for the healing process and re-epithelization of the wound site^25^. Thus, epithelial cell proliferation and angiogenesis are crucial for the healing process^26^. Habibipour *et al*.^27^ showed that treated wounds had a high degree of fibroblast proliferation, collagen synthesis and neovascularization in their histological analysis. This increased wound tensile strength and accelerated wound healing, findings that are comparable with our western blot results on cluster of differentiation 31 (CD31) protein expression, which was increased in the groups treated with CMLA or Intrasite gel; suggesting well-formed blood vessels in these groups.

The antimicrobial features of the CMLA may improve the rate of the wound healing process by promoting wound contraction and increasing the rate of epithelialization^28^. Moreover, CMLA can reduce lipid peroxidation by blocking and slowing the onset of cell necrosis and improving vascularity. This findings confirm that any treatment capable of preventing lipid peroxidation can improve the viability of collagen fibers by improving their strength, improving circulation, preventing cell damage and promoting DNA synthesis^29^.

CMLA has demonstrated antioxidant activity and previous studies have revealed that the production of antioxidants in the wound area enables wound healing^30^. Antioxidants have wound healing properties and can protect tissues from oxidative damage^31^. CMLA possesses antioxidant activity as it increased the activities of antioxidant enzymes in tissue homogenates. The reactive intermediates from oxidation could delay wound healing^15^. Moreover, wound healing may be accelerated by the anti-inflammatory properties of CMLA.

In this study, tissue homogenates from wounds that were treated with CMLA demonstrated notable antioxidant activity with a decrease in the level of MDA and an increase in the levels of GPx and SOD. Free radicals and reactive oxygen species (ROS) are constantly formed by the human body; ROS mediate cell damage. Intercellular and extracellular antioxidants are thus important to protect tissues from oxidative injury^2^. Superoxide dismutase (SOD) converts superoxide to hydrogen peroxide (H_2_O_2_), which is then converted into water by catalase in lysosomes or by glutathione peroxidase (GPx) in mitochondria^32^. Wound homogenates treated with gum acacia showed reduced SOD and GPx activities, which could have been caused by an increase in the production of ROS, which can curb the activity of these enzymes^33^. Topical application of CMLA enhanced the activities of these enzymes and prevented the harmful effects of free radicals generated in rats. Superoxide and hydroxyl radicals are vital mediators of oxidative stress that contribute to clinical disorders. Therefore, any natural or synthetic element with antioxidant properties can totally or partially reduce the harm caused by free radicals^34^. The removal of superoxide and hydroxyl radicals can help to defend an organism from disease. Reductions in the activities of SOD and GPx were observed in the tissue homogenates of control rats. This might have resulted from the decomposition of superoxide anions generated by lipid peroxidation. Reduced activities of these enzymes may result in several harmful effects. Dietary GPx can suppress oxidative stress *in vivo*^35^. Lipid peroxidation is an important pathophysiological event in several diseases^36^. MDA from lipid peroxidation can interact with DNA bases and induce mutagenic lesions^37^. Pratibha *et al*.^38^ showed that activated oxygen species can stimulate cellular activities such as enzyme inactivation, DNA strand cleavage and membrane lipid peroxidation, which are harmful to normal cells.

Heat shock protein 70 (Hsp70) is a protein from the heat shock proteins (HSP) family. It is found in the cells of all mammals and accelerate wound healing by upregulating macrophage-mediated phagocytosis^39^. In addition to that, Hsp70 has the capability to protect the cells from oxidative stress as well as heat shock, and has been associated with proliferation in tissue. The appearance of Hsp70 depends on the location, the number of fibroblasts and the density of collagen fibers. Nevertheless, the differences noted in healed wound tissues might be due to the wound environment as the healing process progresses in granulation tissue from the wound margin to its center^40^. In the present study, groups treated with CMLA showed increased Hsp70 staining, suggesting that CMLA accelerates the wound-healing process.

Bcl-2-associated X protein (Bax) is a pro-apoptotic protein that promotes apoptosis^41^, while B-cell lymphoma 2 (Bcl-2) is an anti-apoptotic protein that impedes this process. These proteins preserve the structure of normal proteins and repair or remove damaged proteins^41^. They are also vital in maintaining cellular homeostasis despite alterations in physiological and environmental factors^42^. Outcomes from the current study revealed that, Bax staining was decreased in the groups topically treated with CMLA or Intrasite gel, while rats treated with gum acacia showed increased Bax staining; this may indicate increased apoptosis which would decrease tissue cellularity in these animals. Moreover, our findings show that there was a delay in apoptosis in the wounds treated with gum acacia as the wounds remained open for a longer period of time. Prolonged exposure to the external influences will increase the risk of inflammation, which will persist until the wound is completely healed.

The use of transforming growth factor (TGF-β) to improve the healing of poorly vascularized or non-healing wounds in the lower equine limb and in immunocompromised, diabetic or elderly individuals could allow for better healing of acute wounds and lead to limb salvage, which would be a significant personal, economic and social advantage^33^. In the current study, it is possible that TGF-β was available in the extravascular environment and thus may have been capable of priming cells, leading to increased responsiveness to normal regulatory factors at the site of injury. This was noted in the treated groups and may have been facilitated by the presence of CMLA.

One of the important findings in this experiment was that CMLA did not trigger any pain or irritation in the subjects. The rats did not show any signs of biting or scratching in the wound area where the CMLA was applied. They also did not display any signs of restlessness. As for the surgical process used to create the wounds, treatment and dressing were performed under sterile conditions. Wounds that displayed any signs of infection were excluded from this study.

## Conclusion

CMLA revealed remarkable wound healing properties. The histology results on day 15 after wound creation revealed less scarring of the wound area. The granulation tissue contained fewer inflammatory cells, as well as more collagen, fibroblasts and capillaries when compared to the wounds of rats treated with gum acacia. Furthermore, CMLA significantly increased antioxidant defense enzyme (GPx and SOD) activity in wound homogenates and decreased MDA levels. These findings were confirmed by immunohistochemistry, which revealed the upregulation of Hsp70 along with TGF-β and the downregulation of Bax proteins.

## Additional Information

**How to cite this article**: El-Ferjani, R. M. *et al. In vivo* Assessment of Antioxidant and Wound Healing Improvement of a New Schiff Base Derived Co (II) Complex in Rats. *Sci. Rep.* **6**, 38748; doi: 10.1038/srep38748 (2016).

**Publisher's note:** Springer Nature remains neutral with regard to jurisdictional claims in published maps and institutional affiliations.

## Figures and Tables

**Table 1:** Elemental analysis and spectral characterization for the ligand and its metal complex.

Ligand Elemental Analysis	Analytical Calculated: C, 64.07; H, 6.79; N, 13.59.
	Found: C, 64.02; H, 6.75; N, 13.53.
IR (KBr)v(OH), 3397; v(C = N), 1576; v(C OO−)1506.	
UV-Vis (DMSO), λ max (€ Mol^−1^ cm^−1^):	280 nm (9333,(***π*** → π*); 343 nm (11433, (n → π*)
1H-NMR (DMSO-d6) (s, 1H, azomethyne), 8.45 (s, 1H, OH)	3.044 (s, 6H, CH3), 7.50–7.24 (4H, ArH), 7.94
Complex Elemental Analysis	Analytical Calculated: C, 33.93; H, 6.68; N, 7.19
Found: C, 33.90; H, 6.55; N, 7.00	
IR (ATR cm21)	v(OH), 3465; v(C = N), 1611; v(C OO−), 1556v(M-N), 596v(M-O)678.
UV-Vis (DMSO)	300 (***π*** → π*); 380 (n → π*); 520 (d → d*).
1H-NMR (DMSO-d6)3.35 (s, 6H, CH3), 6.78–7.02 (4H, ArH), 8.4 (s, 1H, azmthen),	

**Table 2:** Serum biochemical data for male and female rats intragastrically administered CMLA for 14 days.

Parameter	Sex	Normal	MDLA	MDLA
		0.5% CMC	500 mg/kg	1000 mg/kg
Sodium (mmol/L)	Male	149.75 ± 2.9	151.5 ± 1.6	150.75 ± 1.4
	Female	149.73 ± 0.71	152 ± 3.2	151.5 ± 0.58
Potassium (mmol/L)	Male	5.48 ± 0.23	6 ± 1.7	6.17 ± 1.4
	Female	5.21 ± 0.24	5.5 ± 0.1	5.45 ± 0.3
Chloride (mmol/L)	Male	114.85 ± 2.9	114.5 ± 1.8	113.75 ± 3.1
	Female	111.3 ± 2.1	112.3 ± 1.6	112.5 ± 4.8
Carbon dioxide (mmol/L)	Male	18.52 ± 3.6	19.72 ± 2.2	19.3 ± 2.5
	Female	18.45 ± 1.7	19 ± 3.2	18.1 ± 1.1
Anion gap (mmol/L)	Male	21 ± 0.52	22.25 ± 1.3	22.5 ± 2.9
	Female	22.8 ± 0.2	22.5 ± 3.4	22.8 ± 2.5
Urea nitrogen (mmol/L)	Male	9.03 ± 3.1	9.83 ± 0.7	10.3 ± 0.5
	Female	9.32 ± 2.8	9.2 ± 1.7	9.8 ± 3.3
Creatinine (umol/L)	Male	23.61 ± 4.5	24 ± 3.2	23 ± 3.9
	Female	26.01 ± 4.1	23.5 ± 1.8	23 ± 1.5
Total protein (g/L)	Male	57.5 ± 5.6	55.5 ± 2.8	58.75 ± 1.2
	Female	57.4 ± 0.9	56.5 ± 1.6	57.3 ± 0.2
Albumin (g/L)	Male	13.3 ± 2.7	14.25 ± 1.9	14 ± 1.8
	Female	12.5 ± 1.7	13.8 ± 1.6	12.3 ± 1.3
Globulin (g/L)	Male	44.42 ± 4.7	45 ± 3.1	44.75 ± 2.6
	Female	43.5 ± 2.7	43.8 ± 3.2	43.3 ± 3.9
Total bilirubin (umol/L)	Male	2.92 ± 0.2	2.5 ± 0.1	3 ± 0.9
	Female	2.5 ± 0.4	2.5 ± 0.1	2.5 ± 0.7
Alkaline phosphatase (IU/L)	Male	92.5 ± 8.2	93.25 ± 6.1	92.25 ± 3.3
	Female	85 ± 3.9	85 ± 4.1	86.3 ± 5.8
Alanine aminotransferase (IU/L)	Male	53.56 ± 4.8	52.75 ± 4.6	54.75 ± 5.2
	Female	51.3 ± 5.1	50.5 ± 5.8	50.3 ± 3.7
Aspartate Aminotransferase (IU/L)	Male	191 ± 9.5	192.3 ± 11.7	190.5 ± 12.4
	Female	199.4 ± 6.9	201 ± 9.2	200 ± 14.7

Values are expressed as mean ± SEM. There are no significant differences between groups significant value at P < 0.05.

**Table 3:** Time required for wound healing by CMLA in rats.

Dressing type	Healing time (days) (Mean + S.E.M)
Gum acacia (Vehicle)	20.13 ± 0.44
Intrasit gel (standard control)	14.25 ± 0.25*
CMLA (10 mg/ml)	16.00 ± 0.38*
CMLA (20 mg/ml)	13.13 ± 0.29*

Statistical Analysis of the data were carried out using one way analysis of variance (ANOVA) and Dennett’s post hoc test for average comparison on SPSS 18.0. Mean values ±  SEM were used. Significance was defined as *p < 0.05 compared to Gum acacia.

**Table 4:** Effect of CMLA on percentage (%) of wound healing in experimental rats.

Vehicles	Percentage of wound healing (Mean + S.E.M)		
	5 days	10 days	15 days
Gum acacia (Vehicle)	25.19 ± 0.97	53.29 ± 0.91	82.41 ± 1.05
Intrasit gel (standard control)*	65.59 ± 1.29	75.66 ± 1.15	96.46 ± 0.76
CMLA (10 mg/ml)*	55.98 ± 0.98	72.83 ± 0.93	92.82 ± 0.79
CMLA (20 mg/ml)*	68.35 ± 0.98	80.10 ± 1.19	97.83 ± 0.56

Statistical Analysis of the data were carried out using one way analysis of variance (ANOVA) and Dennett’s post hoc test for average comparison on SPSS 18.0. Mean values ± SEM were used. Significance was defined as *p < 0.05 compared to Gum acacia.

## References

[b1] Ram M. . Deferoxamine modulates cytokines and growth factors to accelerate cutaneous wound healing in diabetic rats. Eur J Pharmacol. 764, 9–21 (2015). 26101070 10.1016/j.ejphar.2015.06.029

[b2] Asadi S. Y. . Effect of green tea (Camellia sinensis) extract on healing process of surgical wounds in rat. Int J Surg. 11, 332–337 (2013). 23459184 10.1016/j.ijsu.2013.02.014

[b3] Frieri M., Kumar K. & Boutin A. Wounds, burns, trauma, and injury. Wound Med. 13, 12–17 (2016).

[b4] Abdul Latif M. . Alocasia denudata Engler treatment enhance open wound healing activities in Wistar rat’s skin. J Ethnopharmacol. 176, 258–267 (2015). 26519202 10.1016/j.jep.2015.10.036

[b5] Zayed E. M. & Zayed M. A. Synthesis of novel Schiff’s bases of highly potential biological activities and their structure investigation. Spectrochim Acta Mol Biomol Spectrosc. 143, 81–90 (2015). 10.1016/j.saa.2015.02.024 25721778

[b6] Abou-Hussein A. A. & Linert W. Synthesis, spectroscopic studies and inhibitory activity against bactria and fungi of acyclic and macrocyclic transition metal complexes containing a triamine coumarine Schiff base ligand. Spectrochim Acta Mol Biomol Spectrosc. 141, 223–232 (2015). 10.1016/j.saa.2015.01.063 25681806

[b7] El-Boraey H. A. & El-Gammal O. A. New 15-membered tetraaza (N4) macrocyclic ligand and its transition metal complexes: Spectral, magnetic, thermal and anticancer activity. Spectrochim Acta Mol Biomol Spectrosc. 138, 553–562 (2015). 10.1016/j.saa.2014.11.015 25531404

[b8] Zayed E. M., Zayed M. A. & El-Desawy M. Preparation and structure investigation of novel Schiff bases using spectroscopic, thermal analyses and molecular orbital calculations and studying their biological activities. Spectrochim Acta Mol Biomol Spectrosc. 134, 155–164 (2015). 10.1016/j.saa.2014.06.014 25016203

[b9] Guéant J.-L. . Molecular and cellular effects of vitamin B12 in brain, myocardium and liver through its role as co-factor of methionine synthase. Biochimie 95, 1033–1040 (2013). 23415654 10.1016/j.biochi.2013.01.020

[b10] Ahmad M. . Synthesis and structure elucidation of a cobalt(II) complex as topoisomerase I inhibitor: *In vitro* DNA binding, nuclease and RBC hemolysis. Eur J Med Chem. 74, 683–693 (2014). 24287075 10.1016/j.ejmech.2013.10.025

[b11] Ghosh K., Mohan V., Kumar P. & Singh U. P. DNA binding, nuclease and superoxide scavenging activity studies on mononuclear cobalt complexes derived from tridentate ligands. Polyhedron 49, 167–176 (2013).

[b12] Abu-Dief A. M. & Mohamed I. M. A. A review on versatile applications of transition metal complexes incorporating Schiff bases. Beni-Seuf Univ J Appl Sci. 4, 119–133 (2015). 10.1016/j.bjbas.2015.05.004 PMC7104041 32289037

[b13] Tabrizi L., McArdle P., Erxleben A. & Chiniforoshan H. Nickel(II) and cobalt(II) complexes of lidocaine: Synthesis, structure and comparative *in vitro* evaluations of biological perspectives. Eur J Med Chem. 103, 516–529 (2015). 26402729 10.1016/j.ejmech.2015.09.018

[b14] Heilmann S. . A thermosensitive morphine-containing hydrogel for the treatment of large-scale skin wounds. Int J Pharm. 444, 96–102 (2013). 23352858 10.1016/j.ijpharm.2013.01.027

[b15] Ammar I. . Antioxidant, antibacterial and *in vivo* dermal wound healing effects of Opuntia flower extracts. Int J Biol Macromol. 81, 483–490 (2015). 26306411 10.1016/j.ijbiomac.2015.08.039

[b16] Ben-saber S. M., Maihub A. A., Hudere S. S. & El-ajaily M. M. Complexation behavior of Schiff base toward transition metal ions. Microchem J. 81, 191–194 (2005).

[b17] Ibrahim M. Y. . *α*-Mangostin from Cratoxylum arborescens: An *in vitro* and *in vivo* toxicological evaluation. Arabian J Chem. 8, 129–137 (2015).

[b18] Ibrahim M. Y. . Acute toxicity and gastroprotection studies of a new schiff base derived Manganese (II) complex against Hcl/Ethanol-induced gastric ulcerations in rats. Sci Rep 6, 26819 (2016). 27229938 10.1038/srep26819 PMC4882520

[b19] Nayak B. & Pereira L. M. P. Catharanthus roseus flower extract has wound-healing activity in Sprague Dawley rats. BMC Complement Altern Med. 6, 41–50 (2006). 17184528 10.1186/1472-6882-6-41 PMC1764761

[b20] Kumar M., Prasad S. K., Krishnamurthy S. & Hemalatha S. Antihyperglycemic activity of Houttuynia cordata Thunb. in streptozotocin-induced diabetic rats. Adv Pharmacol Sci. 6, 41–50 (2014). 10.1155/2014/809438 PMC3953599 24707284

[b21] Wells A., Nuschke A. & Yates C. C. Skin tissue repair: Matrix microenvironmental influences. Matrix Biol. 49, 25–36 (2016). 26278492 10.1016/j.matbio.2015.08.001 PMC4753148

[b22] Mughrabi F. . Effect of bis [benzyl N′-(indol-3-ylmethylene)-hydrazinecarbodithioato]-zinc (II) derivatives on wound healing in Sprague Dawley rats. Indian J Exp Biol. 49, 50–55 (2011). 21365996

[b23] Silva J. P. . Improved burn wound healing by the antimicrobial peptide LLKKK18 released from conjugates with dextrin embedded in a carbopol gel. Acta Biomater. 26, 249–262 (2015). 26234490 10.1016/j.actbio.2015.07.043

[b24] Kováč I. . Plantago lanceolata L. water extract induces transition of fibroblasts into myofibroblasts and increases tensile strength of healing skin wounds. J Pharm Pharmacol. 67, 117–125 (2015). 25244603 10.1111/jphp.12316

[b25] Ram M. . Bilirubin modulated cytokines, growth factors and angiogenesis to improve cutaneous wound healing process in diabetic rats. Int J Immunopharmacol. 30, 137–149 (2016). 10.1016/j.intimp.2015.11.037 26679676

[b26] Greaves N. S., Ashcroft K. J., Baguneid M. & Bayat A. Current understanding of molecular and cellular mechanisms in fibroplasia and angiogenesis during acute wound healing. J Dermatol Sci. 72, 206–217 (2013). 23958517 10.1016/j.jdermsci.2013.07.008

[b27] Habibipour S. . Effect of sodium diphenylhydantoin on skin wound healing in rats. Plast Reconstr Surg. 112, 1620–1627 (2003). 14578793 10.1097/01.PRS.0000086773.96319.DA

[b28] Gan C.-Y. & Latiff A. A. Optimisation of the solvent extraction of bioactive compounds from Parkia speciosa pod using response surface methodology. Food Chem. 124, 1277–1283 (2011).

[b29] George B. P., Parimelazhagan T. & Chandran R. Anti-inflammatory and wound healing properties of Rubus fairholmianus Gard. Root: An *in vivo* study. Ind Crops Prod. 54, 216–225 (2014).

[b30] Saltman A. E. d-ribose-l-cysteine supplementation enhances wound healing in a rodent model. Am J Surg. 210, 153–158 (2015). 25935230 10.1016/j.amjsurg.2014.11.014

[b31] Dastmalchi K., Wang I. & Stark R. E. Potato wound-healing tissues: A rich source of natural antioxidant molecules with potential for food preservation. Food Chem. 210, 473–480 (2016). 27211673 10.1016/j.foodchem.2016.04.123 PMC4893199

[b32] Johansen J. S., Harris A. K. & Rychly D. J. Oxidative stress and the use of antioxidants in diabetes: Linking basic science to clinical practice. Cardiovasc Diabetol. 4, 5–12 (2005). 15862133 10.1186/1475-2840-4-5 PMC1131912

[b33] Akbik D., Ghadiri M., Chrzanowski W. & Rohanizadeh R. Curcumin as a wound healing agent. Life Sci. 116, 1–7 (2014). 25200875 10.1016/j.lfs.2014.08.016

[b34] Silveira P. C. L. . Iontophoresis with gold nanoparticles improves mitochondrial activity and oxidative stress markers of burn wounds. Mater Sci Eng C. 44, 380–385 (2014). 10.1016/j.msec.2014.08.045 25280718

[b35] Sudsai T., Wattanapiromsakul C. & Tewtrakul S. Wound healing property of isolated compounds from Boesenbergia kingii rhizomes. J Ethnopharmacol. 184, 42–48 (2016). 26945979 10.1016/j.jep.2016.03.001

[b36] Chigurupati S. . Effects of cerium oxide nanoparticles on the growth of keratinocytes, fibroblasts and vascular endothelial cells in cutaneous wound healing. Biomaterials 34, 2194–2201 (2013). 23266256 10.1016/j.biomaterials.2012.11.061 PMC3552035

[b37] Gonçalves R. V. . 5α-Dihydrotestosterone enhances wound healing in diabetic rats. Life Sci. 152, 67–75 (2016). 27009546 10.1016/j.lfs.2016.03.019

[b38] Pratibha R., Sameer R., Rataboli P. V., Bhiwgade D. A. & Dhume C. Y. Enzymatic studies of cisplatin induced oxidative stress in hepatic tissue of rats. Eur J Pharmacol. 532, 290–293 (2006). 16458885 10.1016/j.ejphar.2006.01.007

[b39] Atalay M. . Heat shock proteins in diabetes and wound healing. Curr Protein Pept Sci. 10, 85–95 (2009). 19275675 10.2174/138920309787315202 PMC2743605

[b40] Bellaye P.-S., Burgy O., Causse S., Garrido C. & Bonniaud P. Heat shock proteins in fibrosis and wound healing: Good or evil? Pharmacol Ther. 143, 119–132 (2014). 24582969 10.1016/j.pharmthera.2014.02.009

[b41] Tan J.-Q. . The roles of autophagy and apoptosis in burn wound progression in rats. Burns 39, 1551–1556 (2013). 23751274 10.1016/j.burns.2013.04.018

[b42] Cui Y. . Relationship between expression of Bax and Bcl-2 proteins and apoptosis in radiation compound wound healing of rats. Chin J Traumatol. 6, 135–138 (2003). 12749782

